# Gain of UBE2D1 facilitates hepatocellular carcinoma progression and is associated with DNA damage caused by continuous IL-6

**DOI:** 10.1186/s13046-018-0951-8

**Published:** 2018-11-27

**Authors:** Chuanchuan Zhou, Fengrui Bi, Jihang Yuan, Fu Yang, Shuhan Sun

**Affiliations:** 1 Department of Medical Genetics, Second Military Medical University, Shanghai, 200433 China; 2 Reproductive Medicine Center, Sixth Affiliated Hospital of Sun Yat-sen University, Guangzhou, 510000 China

**Keywords:** UBE2D1, Hepatocellular carcinoma, Copy number variations, Continuous IL-6, RAD51B

## Abstract

**Background:**

Hepatocellular carcinoma (HCC) is the most common type of liver cancer with increasing incidence and poor prognosis. Ubiquitination regulators are reported to play crucial roles in HCC carcinogenesis. UBE2D1, one of family member of E2 ubiquitin conjugating enzyme, mediates the ubiquitination and degradation of tumor suppressor protein p53. However, the expression and functional roles of UBE2D1 in HCC was unknown.

**Methods:**

Immunohistochemistry (IHC), western blotting, and real-time PCR were used to detect the protein, transcription and genomic levels of UBE2D1 in HCC tissues with paired nontumor tissues, precancerous lesions and hepatitis liver tissues. Four HCC cell lines and two immortalized hepatic cell lines were used to evaluate the functional roles and underlying mechanisms of UBE2D1 in HCC initiation and progression in vitro and in vivo. The contributors to UBE2D1 genomic amplification were first evaluated by performing a correlation analysis between UBE2D1 genomic levels with clinical data of HCC patients, and then evaluated in HCC and hepatic cell lines.

**Results:**

Expression of UBE2D1 was significantly increased in HCC tissues and precancerous lesions and was associated with reduced survival of HCC patients. Upregulation of UBE2D1 promoted HCC growth in vitro and in vivo by decreasing the p53 in ubiquitination-dependent pathway. High expression of UBE2D1 was attributed to the recurrent genomic copy number gain, which was associated with high serum IL-6 level of HCC patients. Further experiments showed that continuous IL-6 activated the DNA damage response and genomic instability by repressing DNA damage checkpoint protein RAD51B. Moreover, continuous IL-6 could significantly facilitate the HCC growth especially with the genomic gain of UBE2D1.

**Conclusions:**

Our findings showed that UBE2D1 played a crucial role in HCC progression, and suggested a novel pattern of continuous IL-6 to promote cancers by inducing the genomic alterations of specific oncogenes.

**Electronic supplementary material:**

The online version of this article (10.1186/s13046-018-0951-8) contains supplementary material, which is available to authorized users.

## Background

Hepatocellular carcinoma (HCC) is one of the most common and fatal human cancers with increasing incidence and mortality worldwide, especially in East Asia and South Africa [1, 2]. Although several risk factors have been identified to be responsible for the HCC initiation, such as hepatitis B virus (HBV) and hepatitis C virus (HCV) infections, alcohol abuse and obesity, the diagnose of HCC at an early stage is still difficult to realize [3]. And advanced stage HCC patients can only benefit from the systemic treatment of sorafenib, which will lose efficacy due to inherent or acquired drug resistance [4]. Therefore, it is important to unravel the molecular events underlying HCC initiation and to identify new therapeutic targets.

Recent studies have identified a series of regulators to mediate the ubiquitination of tumor suppressor genes to promote HCC growth and carcinogenesis [5, 6]. UBE2D1, a member of E2 ubiquitin conjugating enzyme, belongs to the UBE2D family. It has been reported that UBE2D1 mediates the ubiquitination and degradation of tumor suppressor protein p53 in vitro and in vivo [7–10], and UBE2D1 participates the ubiquitination of HSP90AB1, which contributes to the stability and transport to nucleus of p53 [11, 12]. Recently, UBE2D1 was reported to connect the overexpression of Aurora kinase A with Wnt and Ras-MAPK signaling pathways in colorectal cancer, suggesting UBE2D1 may play an important role in the progression from adenoma-to-carcinoma [13]. However, the functional roles of UBE2D1 in HCC were unclear.

Copy number variations(CNVs), as one of the important components of genomic variations in human, are closely associated with many genetic and developmental disorders [14, 15]. Differently expressed tumor suppressor genes and oncogenes due to the CNVs were regarded as the crucial cause of many types of cancer, including HCC [16, 17]. Genome instability and abnormal DNA damage are the hallmarks of cancers [18, 19]. And previous studies have implicated that DNA damage response and genomic instability would drive the copy number variants formation and common fragile sites arising [20, 21]. Physical damage such as ionizing radiation, and many chemical agents can induced the DNA damage response and genomic instability [22], while HBV integration events might increase the CNVs frequency via inducing genomic instability [19]. Actually, the factors driving DNA damage response and genomic alteration in tumor microenvironment were unclear.

In our study, we explored the roles of UBE2D1 in HCC. UBE2D1 was significantly up-regulated in HCC tissues, and high expression level of UBE2D1 could act as a poor prognosis factor for overall survival of HCC patients. UBE2D1 facilitated the HCC growth in vitro and in vivo by decreasing the p53 protein level in an ubiquitin-dependent manner. We also found that the high expression of UBE2D1 was associated with recurrent genomic gain in HCC. Next, we confirmed the gain of UBE2D1 was attributed to the high IL-6 level, and continuous IL-6 could activate the DNA damage response and genomic instability to drive genomic alterations. From our findings, we identified the IL-6/RAD51B/UBE2D1 axis, suggesting continuous IL-6 as a factor to induce the genomic alterations of oncogenes in HCC carcinogenesis.

## Methods

### Cell culture and treatment

LO2, QSG -7701, SMMC -7721, LM3, Hep3B and Huh7 cells were achieved from the Cell Bank of the Chinese Academy of Sciences (Shanghai, China) and cultured in Dulbecco’s modified Eagle’s medium with 10% fetal bovine serum at 37 °C, 5% CO2. Where indicated, cells were treated with IL-6 (PeproTech, Rocky Hill, NJ) for the indicated time. Without indications, cells were treated with IL-6 at 1 ng/ml for 48 h. IL-6-LT cells were treated with 1 ng/ml IL-6 for more than two months with IL-6 replenishment every 2 days.

### Western blot analysis

Total cell and tissues protein extracts were separated by SDS-PAGE, transferred to nitrocellulose membrane and incubated with antibodies specific for UBE2D1(Abcam, Hong Kong, China), p53(Sigma-Aldrich, Saint Louis, MO), CHK1(Cell Signaling Technology, Boston, USA), phosphorylated CHK1(Cell Signaling Technology), RPA(Sigma-Aldrich), γ-H2Ax(Cell Signaling Technology), STAT3 and phosphorylated STAT3(Cell Signaling Technology), RAD51B(Abcam), cleaved PARP, Caspase 9 and Caspase 3 (Cell Signaling Technology) and β-actin (Sigma-Aldrich). IRdye800-conjugated goat anti-rabbit IgG and IRdye700-conjugated goat anti-mouse IgG were then incubated with the blots. Antibody binding was detected on Odyssey infrared scanner (Li-Cor Biosciences Inc.).

### Immunohistochemistry (IHC)

Immunohistochemistry of UBE2D1 was performed on 5 μm paraffin sections of tissue samples. UBE2D1 antibody (Abcam) was applied at 1:100 dilution. Vectastain Elite ABC kits (Vector, Burlingame, CA, USA) and ImmPACT DAB Substrate (Vector) were used to detect UBE2D1 expression. The staining sections were visualized in Olympus microscopy (IX71; Olympus, Japan).The score was evaluated as four levels:0 (−), 1(+), 2(++), 3(+++). The quantitative analyses of immunohistochemistry images were performed using Image Software Pro Plus 6.0.

### Lentivirus mediated the overexpression of target genes

To obtain the overexpression of UBE2D1, STAT3 and RAD51B, the indicated cells were transfected with recombinant lentiviruses. Recombinant lentiviruses containing UBE2D1, STAT3 and RAD51B with negative control were purchased from Obio Technology (Shanghai, China). For transfection of UBE2D1, STAT3 and RAD51B into indicated cells, 1 × 10^6^ transducing units were added to the medium of cultured cells, and supernatant was replaced with complete culture media after 24 h. The overexpression of genes was confirmed by real-time PCR and Western blot with specific primers or antibodies 96 h after infection. For construction of stably overexpressed UBE2D1, STAT3 and RAD51B, the cells were transfected with 1 × 10^6^ transducing units’ lentivirus, and the transfected cells were selected by using puromycin at the concentration of 0.5 μg/ml to 1.0 μg/ml for indicated cells. The overexpression of target genes was confirmed by real-time PCR and Western blot.

### RNA interference

UBE2D1 and STAT3 interference was mediated by siRNA. RAD51B interference experiments were performed using shRNA vector (genechem, shanghai, China). For transfections, 75 nM siRNA or 4 μg shRNA and negative controls were transfected into indicated cells with Lipofectamine 3000 (Invitrogen). The oligonucleotide sequences of siRNAs were presented in Additional file 1: Table S3.

### Cell-counting kit-8 (CCK-8)assays

Cells were incubated in normal culture medium with 10 μl CCK-8 (Dojindo, Kumamoto, Japan) in 96-wellplate for 2 h. Then the cell numbers were estimated by value of optical density (OD) at 450 nm. The cell proliferation rates were determined at 0, 12, 24, 48, 60 and 72 h after treatment.

### Plate clone formation assays

Indicated cells were cultured in 6-well plate with 500 cells per well. Then the plates were incubated at 37 °C with 5% CO_2_ for 14 days with medium renewal every 4 days. After being fixed with 3.7% formaldehyde for 20 min, the cells were stained with 0.5% Crystal violet dye.

### Immunofluorescence analysis

Cells for immunofluorescence analysis were prepared on 12 × 12 mm glass slides in 12-well plate. The cells were firstly incubated with specific antibodies of γ-H2Ax(Cell Signaling Technology), p53(Abcam), UBE2D1(Abcam) and then the goat anti -rabbit IgG (Alexa Fluor 594, Invitrogen; cy3,sangon) was used as the secondary antibody. The nuclei were stained by DAPI, and images were captured under the Olympus microscopy (IX71; Olympus, Japan).

### TUNEL assays

For the detection of apoptosis, TUNEL staining was performed according to the manufacturer’s instruction (Biotool, Houston,TX,USA). Cells were cultured in slides in 12-well plate, and fixed with 4% paraformaldehyde solution. After permeabilizing slides in 0.2% Triton X-100 solution, cells were incubated with reaction mixture for 1 h. Nuclei were stained by DAPI, and images were captured under the Olympus microscopy (IX71; Olympus, Japan).

### Patients

HCC tissues and paired adjacent noncancerous tissues(at least 3 cm away from tumor boarder) in Cohort, normal hepatic tissues(distal normal liver tissue of liver hemangioma patients), hepatitis liver tissues, precancerous lesions and paired tissues without lesions, serum samples were collected from Eastern Hepatobiliary Surgery Hospital, Shanghai, China (Additional file 1: Table S1 and Table S2). For precancerous lesions, we refers to high grade dysplastic nodules (HGDN), which is defined as nodules with high malignant potential and high grade atypia. The non-lesions tissues were collected from tissues at least 1 cm away from HGDN edge. The HGDN was diagnosed by two pathologists and met the criteria for HGDN reported previously [23]. All patients provided written informed consent, and the experiments were approved by ethics committee of Second Military Medical University.

### Animal studies

All animal experiments were approved by Institutional Animal CareandUse Committee(IACUC) of Second Military Medical University, Shanghai, China. Animals were maintained under standard conditions, in accordance with institutional animal care guidelines. HCC cells with UBE2D1 overexpression and control or continuous IL-6 stimulation were injected subcutaneously into the bilateral armpit of nude mice, and tumors were collected after 3–4 weeks to evaluate the tumor growth. As cells with UBE2D1 overexpression or continuous IL-6 stimulation and control cells were injected subcutaneously into the left and right armpits respectively of the same nude mice, the Wilcoxon signed-rank test for statistical testing was adopted.

### Statistical analysis

All the statistical analyses were performed with SPSS version 21 software. For comparisons, one-way analyses of variance, Wilcoxon signed-rank test and two-tailed Student’s t-test were performed, as appropriate. For analyzing survival of HCC patients, Kaplan-Meier method were used. *P* value < 0.05 was considered significant.

## Results

### UBE2D1 was upregulated in HCC and predicted a poor prognosis of HCC patients

**Fig. 1:**
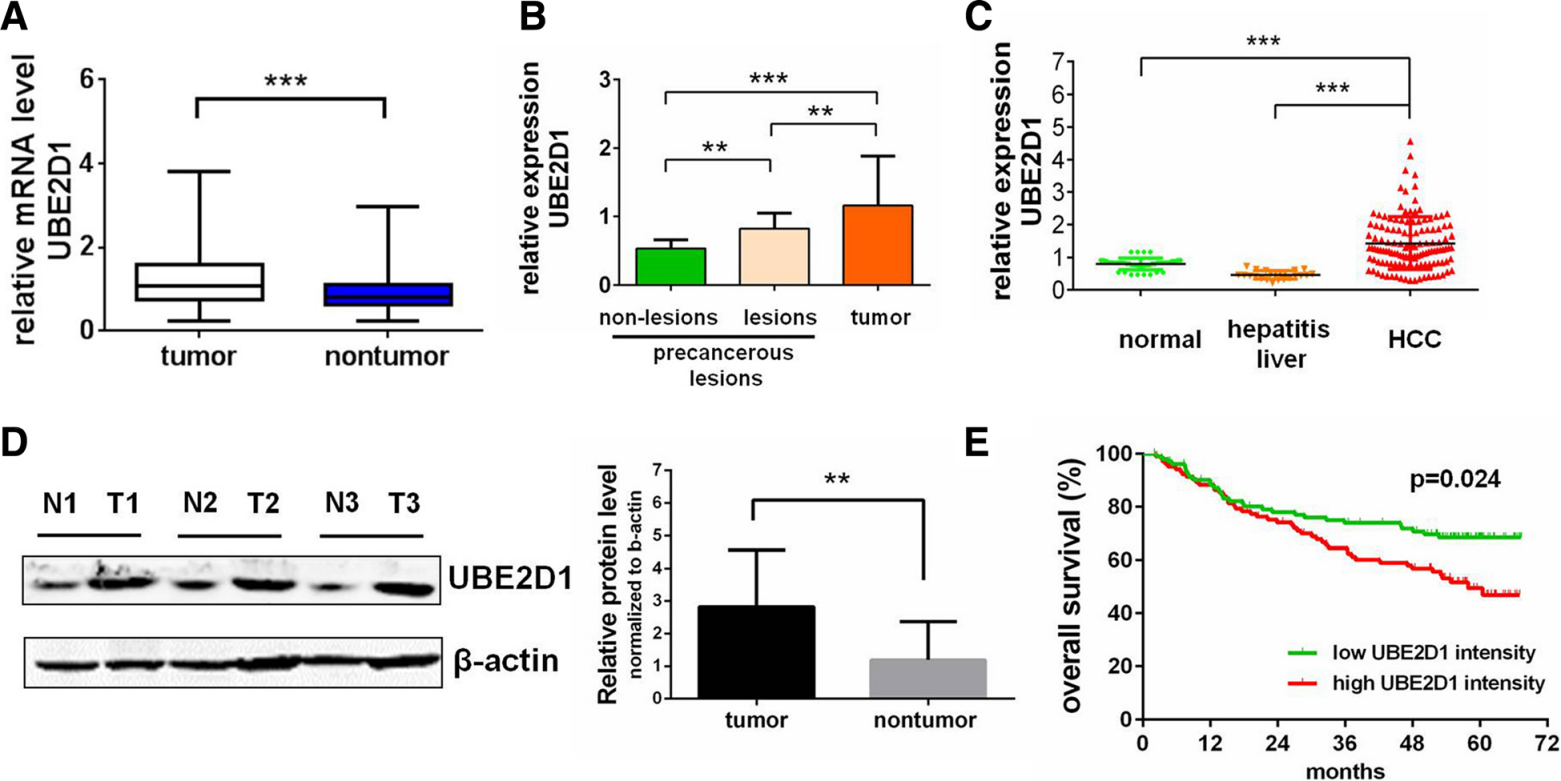
UBE2D1 was upregulated in HCC and predicted a poor prognosis of HCC patients. **a** The transcript level of UBE2D1 were determined by real-time PCR in HCC and paired adjacent nontumor tissues in HCC Cohort (*n* = 108). β-actin was used as internal control. **b** Relative expression of UBE2D1 measured by quantitative PCR in precancerous lesions and paired nontumor tissues without lesions(*n* = 9) and HCC tissues(n = 108). **c** Relative transcript level of UBE2D1 measured by quantitative PCR in normal(*n* = 41) and hepatitis liver tissues(*n* = 22) compared with in HCC tissues. **d** Representative western blotting image of UBE2D1 in tumor(T) and corresponding nontumor tissues(N)(*n* = 19). 12/19, upregulated in tumor; 3/19, downregulated in tumor; 4/19, no differences between tumor and nontumor. The value of blotting images was showed in the right panel. **e** Kaplan-Meier analyses of the correlations between relative intensity of UBE2D1 in GSE14520 and overall survival of HCC patients. The median intensity was used as the cutoff for low and high group. **p < 0.01, ***p < 0.001

To elucidate the roles of UBE2D1 in HCC progression, we detected its expression in HCC tissues. We found that UBE2D1 was significantly upregulated in HCC tissues compared with corresponding noncancerous hepatic tissues in a set of HCC Cohort (*n* = 108) and Cohort from online database(GSE14520, *n* = 209) (Fig. 1a and Additional file 1: Figure S1A). Then we examined the UBE2D1 expression in the precancerous lesions and paired adjacent tissues without lesions to find that precancerous lesions had a higher UBE2D1 level than adjacent normal tissues (Fig. 1b), indicating the pro-tumor role of UBE2D1 at the early stage of HCC. To further confirm the increase of UBE2D1 in hepatocellular carcinogenesis, we also measured its transcript level in normal hepatic tissues and hepatitis liver tissues without HCC to find that the upregulation of UBE2D1 was only in cancer cells but not in hepatitis cells (Fig. 1c), indicating UBE2D1 as a potential early HCC biomarker. Next we confirmed the upregulation of UBE2D1 on the protein level by western blotting and immunohistochemistry (IHC) in HCC (Fig. 1d and Additional file 1: Figure S1B).

To further explore whether UBE2D1 expression level was associated with the clinical outcome of HCC patients, we performed Kaplan-Meier analysis to reveal that the high expression level of UBE2D1 in tumor tissues was correlated with reduced overall survival of the HCC patients in GSE14520 (Fig. 1e). These results demonstrated that UBE2D1 was significantly overexpressed in HCC and premalignant tissues and was a prognostic predictor for HCC patients.

### UBE2D1 promoted HCC growth in vitro and in vivo

**Fig. 2:**
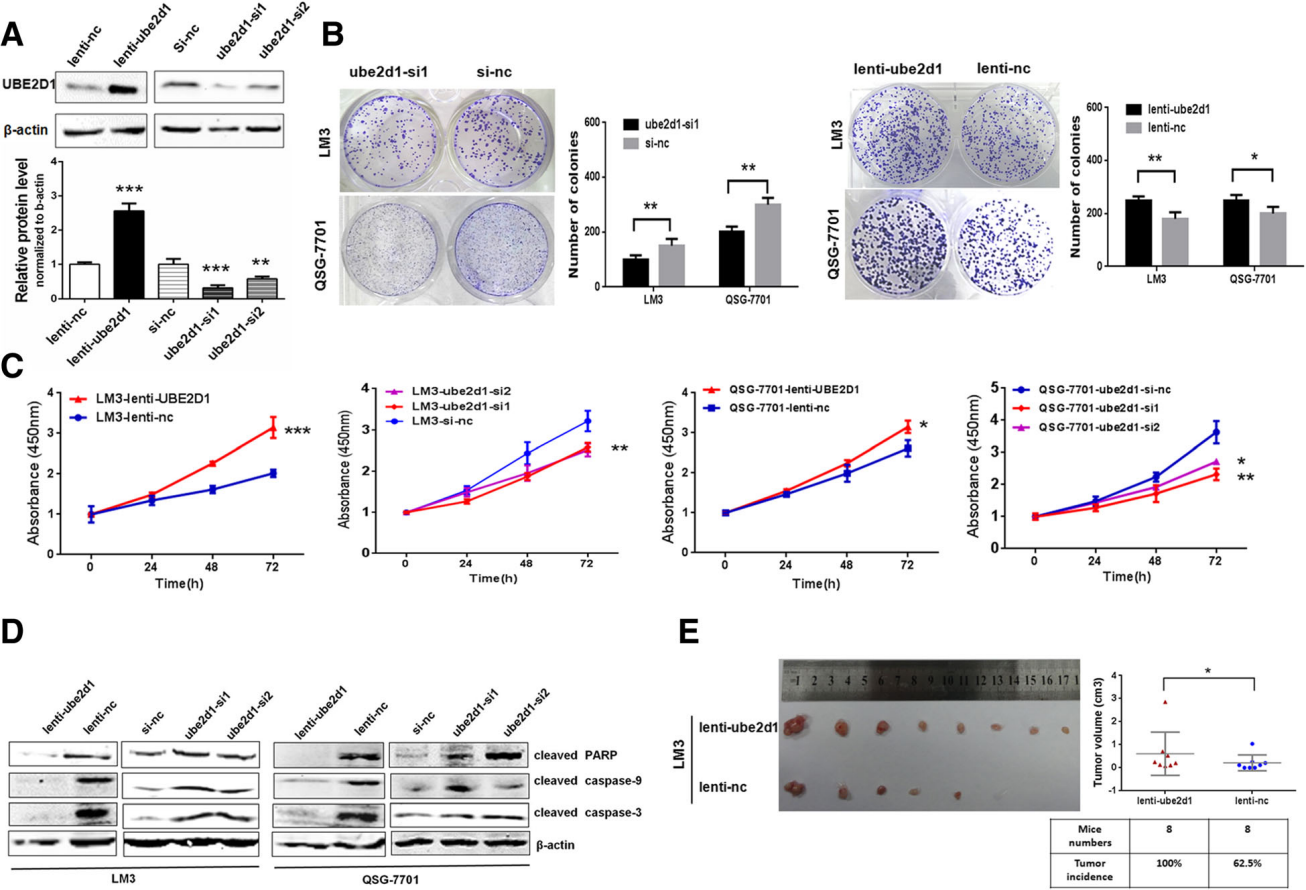
UBE2D1 promoted HCC growth in vitro and in vivo. **a** Confirmation for the overexpression and knockdown of UBE2D1 by western blot. The value of blotting score was showed in the bottom panel. **b** Plate clone formation assays in UBE2D1 overexpressed or underexpressed QSG-7701 and LM3 cells. The number of clones was showed in the right panel. **c** CCK-8 assays for cell proliferation in UBE2D1 overexpressed and silent LM3 and QSG-7701 cells. **d** Apoptosis markers determined by western blot to evaluate the anti-apoptosis role of UBE2D1 in LM3 and QSG-7701 cells. **e** Tumors from nude mice with 1 X 10^5^ indicated cells injected subcutaneously in bilateral armpit. *n* = 8 for each group. And the tumor incidence and tumor volume for each group was showed in the right panel. *p < 0.05,**p < 0.01, ***p < 0.001

We next evaluated the functional roles of UBE2D1 in HCC growth in vitro and in vivo. Firstly, we detected the UBE2D1 abundance in HCC cell lines and normal hepatic cell lines to find that hepatic cells had a lower UBE2D1 level than most of HCC cells (Additional file 1: Figure S2A). Blotting analysis of proteins from nuclear and cytoplasmic fractions revealed that UBE2D1 mainly located in the cytoplasm (Additional file 1: Figure S2B). To determine the role of UBE2D1 in HCC pathogenesis, we conducted the lentivirus-based overexpression and small interfering RNAs (siRNAs)-mediated knockdown (Fig. 2a). We conducted the clone formation assays in HCC cells line LM3 and hepatic cells line QSG-7701 to find that overexpression of UBE2D1 enhanced the clone formation potential, while UBE2D1 knockdown reduced the clone numbers (Fig. 2b). Cell proliferation evaluated by CCK-8 assays was decreased in UBE2D1-silent LM3 and QSG-7701 cells and increased in UBE2D1 overexpressed cells (Fig. 2c and Additional file 1: Figure S2C). Additionally, knockdown of UBE2D1 increased the markers of apoptosis, including cleaved PARP, caspase 3 and caspase 9, while upregulation of UBE2D1 suppressed the apoptosis (Fig. 2d). And the Annexin V-FITC staining confirmed the apoptosis-suppressing role of UBE2D1(Additional file 1: Figure S2D). Moreover, UBE2D1 significantly increased the expression of marker of stem cell-like phenotype and decreased the expression of hepatic specific genes(Additional file 1: Figure S2E). Finally, we explored the role of UBE2D1 in HCC carcinogenesis in vivo by transplanting the UBE2D1 stably-overexpressed cells into the bilateral armpit of nude mice. We found that UBE2D1 could promote the tumor growth in vivo (Fig. 2e).

### P53 mediated the pro-tumor effect of UBE2D1

**Fig. 3:**
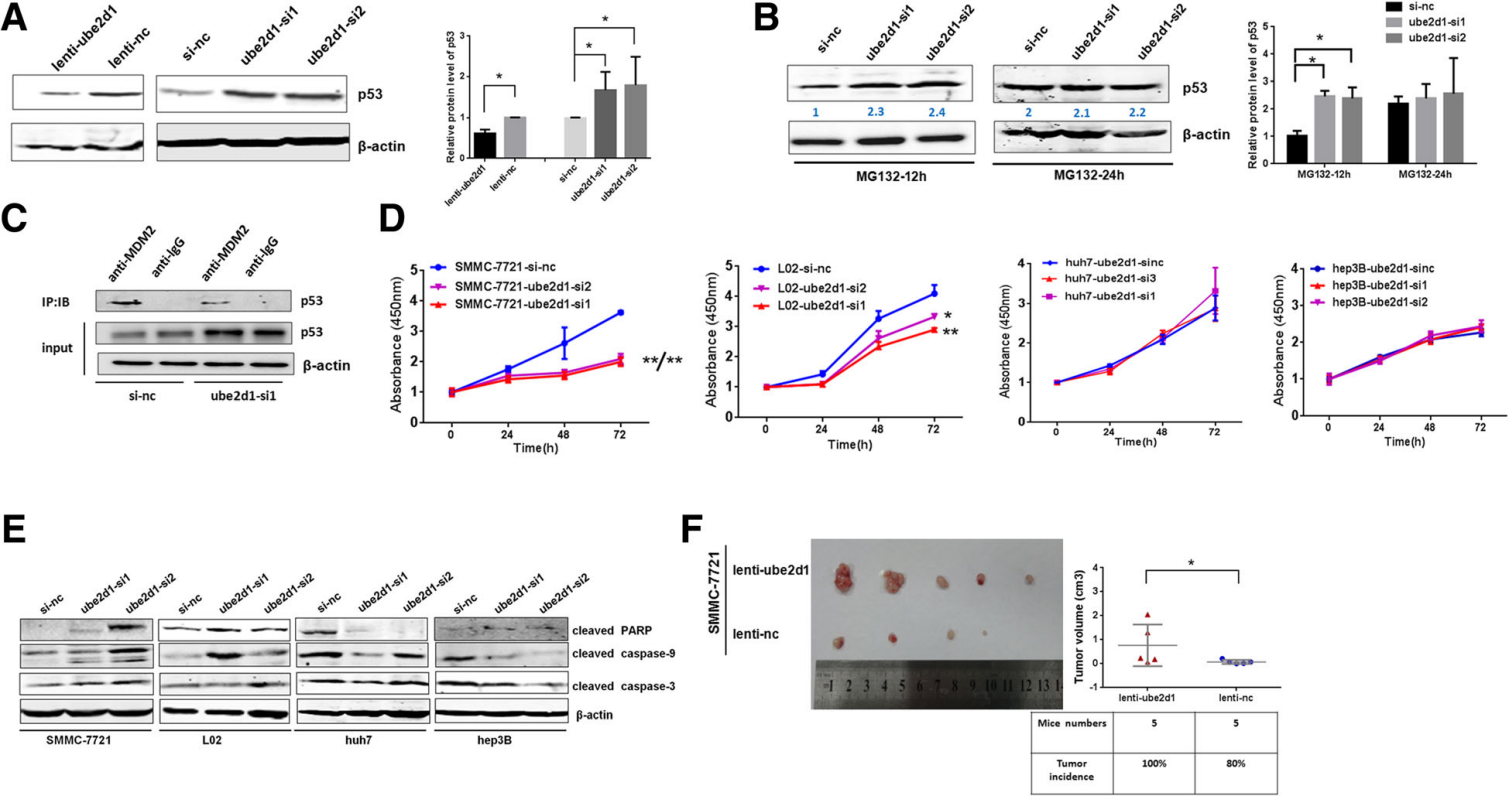
P53 mediated the pro-tumor effect of UBE2D1. **a** p53 protein level determined by western blot in UBE2D1 overexpressed and underexpressed QSG-7701 cells. The quantitation of western blot from 3 independent experiments was showed in the right panel. **b** P53 western blot assays in UBE2D1 silent and negative control QSG-7701 cells after treated with MG132 for 12 h and 24 h. The quantitation of western blot from 3 independent experiments was showed in the right panel. **c** After immunoprecipitation with specific antibodies of MDM2, the purified proteins were detected by western blot in UBE2D1 silent QSG-7701 cells. Immunoprecipitation using mouse lgG antibody was performed as negative control. Input, the whole cell lysate. **d** CCK-8 assays in UBE2D1 underexpressed SMMC-7721, L02, Huh7 and Hep3B cells. **e** Protein levels of apoptosis markers in UBE2D1 underexpressed SMMC-7721, L02, Huh7 and Hep3B cells. **f** Tumors from nude mice with 1 X 10^5^ indicated cells injected subcutaneously in bilateral armpit. *n* = 5 for each group. And the tumor incidence and tumor volume for each group was showed in the right panel. *p < 0.05,**p < 0.01

Since UBE2D1, as an ubiquitin-conjugating enzyme, was reported to be associated with tumor-suppressor protein p53, a key regulator in HCC carcinogenesis, we confirmed the regulation of UBE2D1 on p53 in QSG-7701, LM3 and SMMC-7721 cells. Firstly, we measured the basal expression level of p53 in these three cell lines(Additional file 1: Figure S3A). Next, We found that p53 accumulated following UBE2D1 knockdown, while overexpression of UBE2D1 reduced the p53 protein level (Fig. 3a, Additional file 1: Figure S3B and S3C), Immunofluorescent staining of p53 confirmed the negative regulation of UBE2D1 on p53(Additional file 1: Figure S3D). Meanwhile, p53 transcript level was not affected by UBE2D1 (data not shown). We then treated the UBE2D1 downexpressed and control cells with the proteasome inhibitor MG-132 to block the protein degradation. As a result, MG132 abolished the upregulation of p53 caused by UBE2D1 knockdown in western blot and immunofluorescent staining (Fig. 3b, Additional file 1: Figure S3E and S3F). To further confirm the ubiquitination-dependent degradation of p53 by UBE2D1, we performed the immune-precipitated assays with MDM2 specific antibody in UBE2D1-silent QSG-7701 cells. Results revealed that UBE2D1 knockdown decreased p53-MDM2 interaction(Fig. 3c).

Then we further investigated whether UBE2D1 promoted cell growth through p53 inhibition. As hepatic cell line QSG-7701 and cancer cell line LM3 are p53 wild-type, we examined the cellular functions and mechanisms in other p53 wild-type hepatic cell line L02 and cancer cell line SMMC-7721, p53 mutant-type cancer cell line Huh7 and p53-deficient HCC cell line Hep3B [24]. Results showed that UBE2D1 could promote cell proliferation and repress cell apoptosis in p53 wild-type cell lines, but not in p53 mutated and deficiency cells(Fig. 3d-f and Additional file 1: Figure S3G). Altogether, these results showed that UBE2D1 triggered the degradation of p53 to promote HCC growth.

### Copy number gain contributed to UBE2D1 overexpression and was associated with the high IL-6 level

**Fig. 4:**
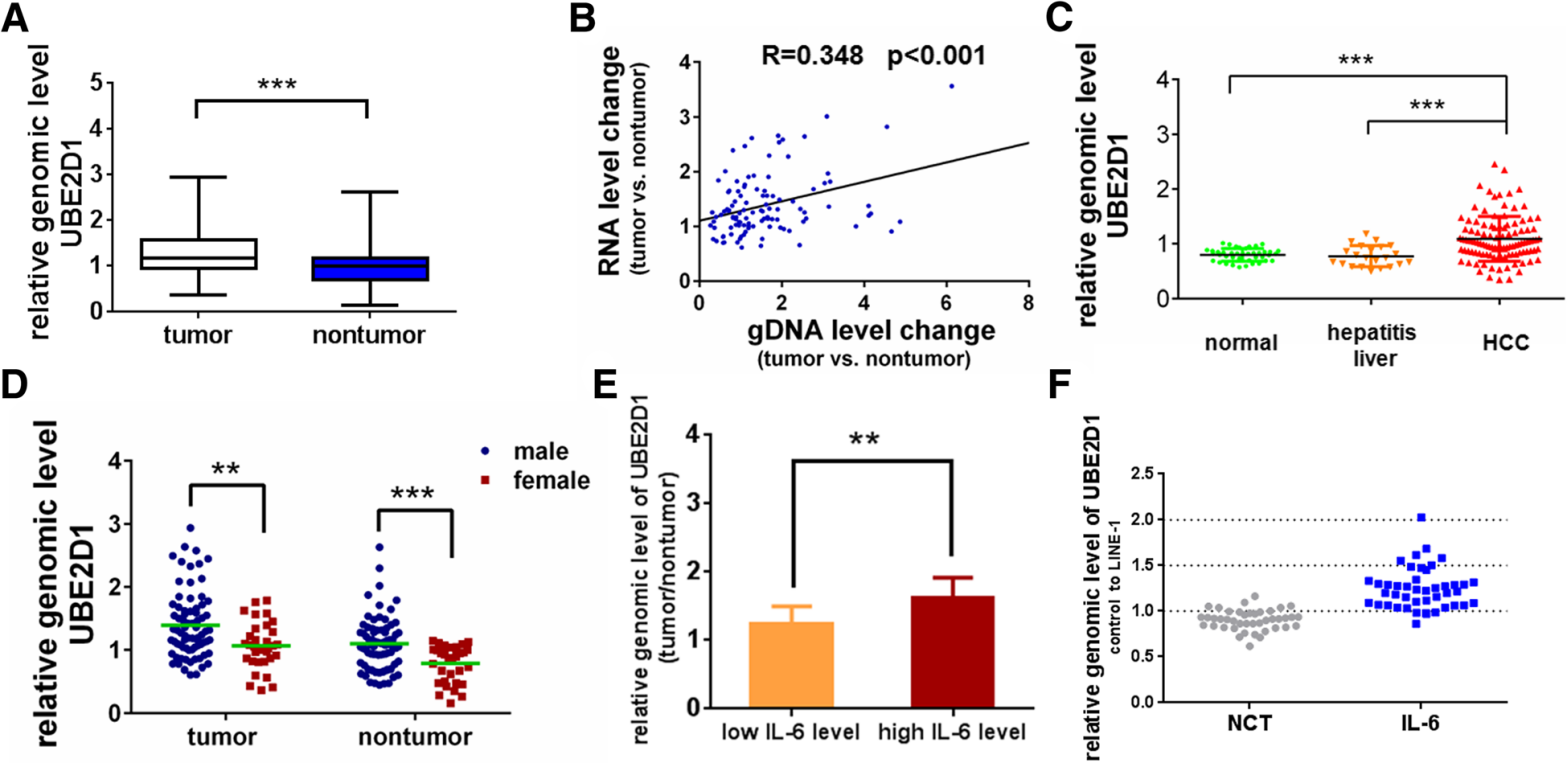
Copy number gain contributed to UBE2D1 overexpression and was associated with the high IL-6 level. **a** The relative genomic level of UBE2D1 were determined by real-time PCR in HCC and paired adjacent nontumor tissues in HCC Cohort (*n* = 108). LINE-1 was used as internal control. **b** Correlation between change on expression levels and genomic levels of UBE2D1. x, the relative UBE2D1 genomic DNA content in HCC compared with nontumor tissues, LINE-1 as an internal control. y, the relative expression level of UBE2D1 in HCC compared with nontumor tissues, β-actin as an internal control. **c** Relative genomic level of UBE2D1 in normal (*n* = 41) and hepatitis liver tissues (*n* = 22) compared with in HCC tissues. **d** The genomic level of UBE2D1 in HCC tissues and adjacent nontumor tissues from female and male HCC patients. **e**The genomic content of UBE2D1 in HCC patients (*n* = 64, from the same set of HCC cohort) with high serum IL-6 and low serum IL-6. y, the relative genomic level of UBE2D1 in HCC compared with nontumor tissues. x, the median intensity was used as the cutoff for low and high group. **f** Genomic levels of UBE2D1 were detected by real-time PCR after continuous IL-6 stimulation in SMMC-7721 cells for more than 4 months. NTC, the negative control cells which was treated with equal amount of pure water(solvent of IL-6). **p < 0.01, ***p < 0.001

As copy number variations is the crucial event early in hepatocarcinogenesis, and controls the gene expression to initiate cancers [25], we investigated whether the upregulation of UBE2D1 in HCC was attributed to copy number variations. By detecting the genomic levels of UBE2D1 in the same set of HCC Cohort, we found that the genome content of UBE2D1 was significantly amplified in HCC and was correlated with its transcriptional level (Fig. 4a, b). Additionally, the HCC tissues had a higher genomic level of UBE2D1 than normal liver tissues or hepatitis liver tissues (Fig. 4c), corresponding to the transcript level. Correlation analysis of UBE2D1 genome content with patients’ information showed that genomic level of UBE2D1 was associated with the gender(Additional file 1: Table S4). Compared with female patients, males had a higher UBE2D1 genomic level both in HCC tissues and adjacent nontumor tissues (Fig. 4d). Gender disparity is a Characteristic for HCC [2], and males are about three to five times more likely to develop HCC than females [26]. The results indicated that the copy number variations of UBE2D1 was associated with gender in HCC, and may be a reason for the gender disparity. Interestingly, we found that the genomic level of UBE2D1 was also associated with the serum IL-6 level of HCC patients, and patients with high serum IL-6 concentration had a higher UBE2D1 genomic level (Fig. 4e). Previous studies have confirmed the crucial roles of IL-6 in the gender disparity of HCC [27, 28]. So we hypothesized that the gender difference of UBE2D1 genome was attributed to the IL-6. To verify this hypothesis, we treated the SMMC-7721 cells with continuous IL-6 for long time to evaluate the genomic alterations of UBE2D1. We found that the genomic content was amplified after continuous IL-6 stimulation for more than 4 months (Fig. 4f). Taken together, the results showed that copy number gain contributed to UBE2D1 overexpression and was associated with the high IL-6 level.

### Continuous IL-6 activated the DNA damage and genomic instability through RAD51B

**Fig. 5:**
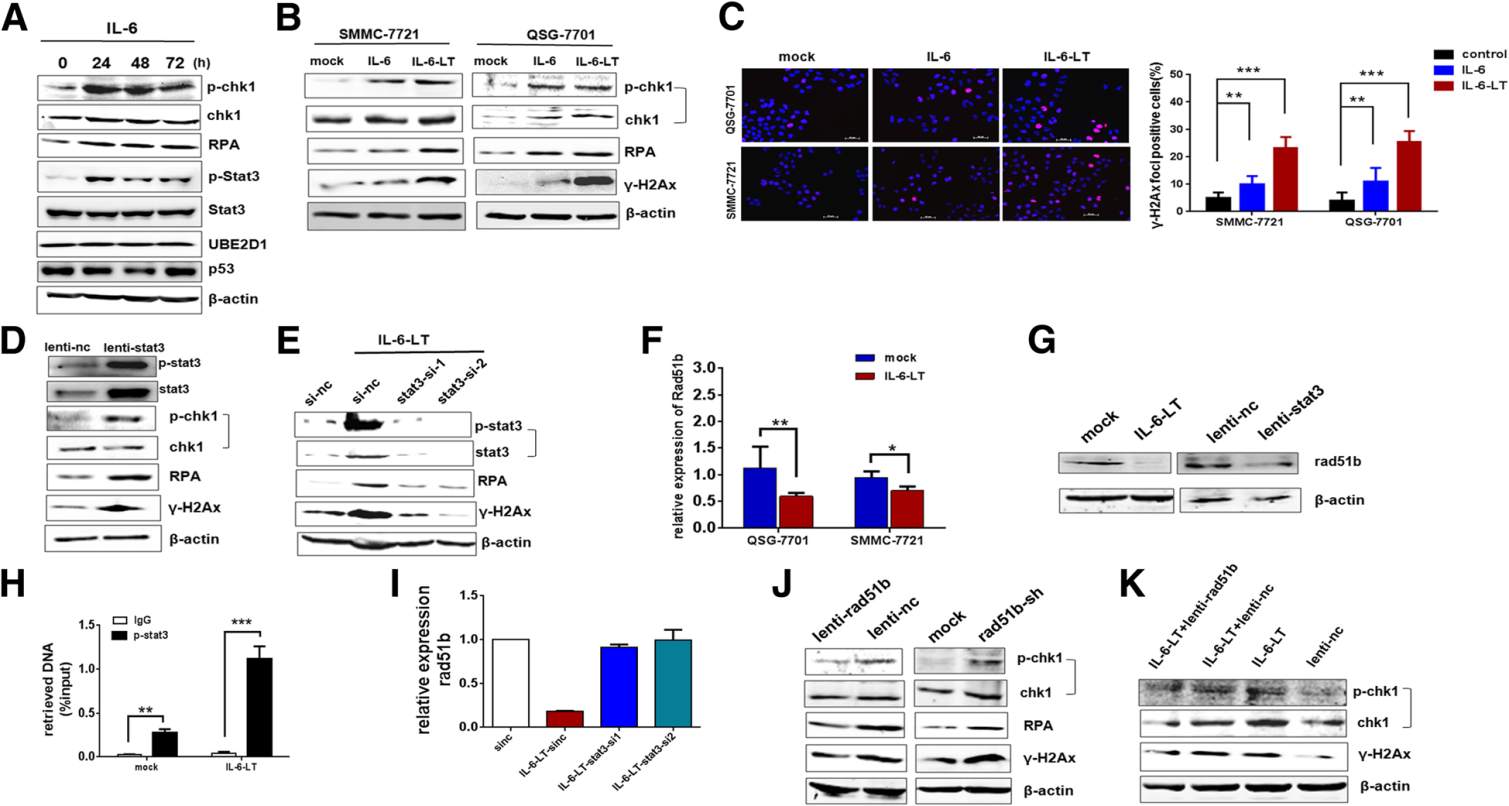
Continuous IL-6 activated the DNA damage and genomic instability through RAD51B. **a** Detection of STAT3, p-STAT3, UBE2D1, p53 and DNA damage marker of p-CHK1 and RPAin IL-6 treated SMMC-7721 cells for 0, 24, 48 and 72 h. **b** DNA damage and genomic instability was detected in continuous IL-6 (more than 2 months) treated QSG-7701 and SMMC-7721 cells. **c** γ-H2Ax foci in QSG-7701 and SMMC-7721 cells treated with IL-6(24 h) and continuous IL-6 were determined by immunofluorescence. Nuclei were stained by DAPI. Scale bar, 50um. The number of γ-H2Ax foci positive cells was showed in the right panel and data was collected from three independent experiments. **d** Protein levels of STAT3, p-STAT3, p-CHK1, RPA and γ-H2Ax in STAT3 overexpressed SMMC-7721 cells. **e** Protein levels of p-CHK1, RPA and γ-H2Ax in STAT3 silent and control SMMC-7721 cells with continuous IL-6. **f** Relative expression of RAD51B in continuous IL-6 treated SMMC-7721 and QSG-7701 cells. **g** Protein level of RAD51B in continuous IL-6 treated and STAT3 overexpressed SMMC-7721 cells. **h** The relative enrichment of p-STAT3 by real-time analysis at the promoter of RAD51B in ChIP assays. The relative enrichment was normalized to corresponding input (whole cell extracts). The primers were designed according to the predict sites of p-STAT3 in the promoter of RAD51B using programs in the gene-regulation. **i** Relative expression of RAD51B in STAT3 silent and control SMMC-7721 cells with continuous IL-6. **j** Protein levels of p-CHK1, RPA and γ-H2Ax in RAD51B overexpressed and silent SMMC-7721 cells. **k** Protein levels of p-CHK1 and γ-H2Ax in RAD51B overexpressed and control SMMC-7721 cells with continuous IL-6. *p < 0.05,**p < 0.01, ***I < 0.001

Next we explored the mechanisms of genomic alterations induced by continuous IL-6 stimulation. As activated DNA damage response and genomic instability induced by exogenous stimulation was the primary reason for genomic alterations in cancer, we detected the effects of IL-6 on DNA damage and genomic instability. IL-6 activated the DNA damage response marked by phosphorylated CHK1 and RPA [29] (Fig. 5a and Additional file 1: Figure. S4A). And continuous IL-6 maintained the activated DNA damage marker and significantly enhanced the genomic instability marked by γ-H2Ax (Fig. 5b). Moreover, continuous IL-6 enhanced the UBE2D1 protein levels and inhibited the p53 expression, but not short-term IL-6 stimulation (Fig. 5a and Additional file 1: Figure S4B). Immunofluorescence of γ-H2Ax foci confirmed the increasing of genomic instability under IL-6 (Fig. 5c). As STAT3 activation is the main event in IL-6 signaling pathway [30], we next performed the lentivirus-mediated STAT3 overexpression to find that STAT3 overexpressed enhanced the DNA damage and genomic instability as continuous IL-6 (Fig. 5d), and interfering STAT3 in continuous IL-6 treated cells abolished the increase of DNA damage and genomic instability caused by IL-6(Fig. 5e). Interestingly, in continuous exogenous IL-6 treated cells, the IL-6 expression was significantly increased, indicating an autocrine IL-6 loop involved in the process (Additional file 1: Figure S4C).

To further explore the underlying DNA damage checkpoint protein regulated by IL-6 and STAT3, we overlapped the STAT3 ChIP-seq data [31, 32] with genes associated with DNA damage repair summarized from previous study. Next we detected these candidate genes in continuous IL-6 treated cells to find that RAD51B was significantly repressed in both QSG-7701 and SMMC-7721 cells with continuous IL-6 stimulation (Fig. 5f and Additional file 1: Figure S4D). And continuous IL-6 could provoke a gradual decline of RAD51B (Additional file 1: Figure S4E). The immunoblotting confirmed the suppression of RAD51B by IL-6 and STAT3 on protein level (Fig. 5g). We then confirmed the genomic binding of STAT3 in the promoter of RAD51B using ChIP with phosphorylated STAT3 specific antibody (Fig. 5h). And the suppressing effect of STAT3 on Rad51b promoter was further confirmed by Rad51b reporter assay (Additional file 1: Figure S4F). Moreover, interfering STAT3 in continuous IL-6 treated cells can abolish the decrease of RAD51B caused by IL-6 (Fig. 5i and Additional file 1: Figure S4G). Next, we detected the functions of RAD51B on DNA damage and genomic instability to find that overexpression of RAD51B could inhibit DNA damage and genomic instability, while RAD51B knockdown enhanced the response (Fig. 5j and Additional file 1: Figure S4H). Restoring the RAD51B expression could rescue the DNA damage response and genomic instability(Fig. 5k), indicating that continuous IL-6 activated the DNA damage and genomic instability through RAD51B. Next, we found that Rad51b stably underexpressed cells had increased UBE2D1 protein levels and decreased p53 expression (Additional file 1: Figure S4I). And long-term Rad51b silent could increase the UBE2D1 genomic content (Additional file 1: Figure S4J).

### Functional roles of IL-6-RAD51B-UBE2D1 axis in HCC

**Fig. 6:**
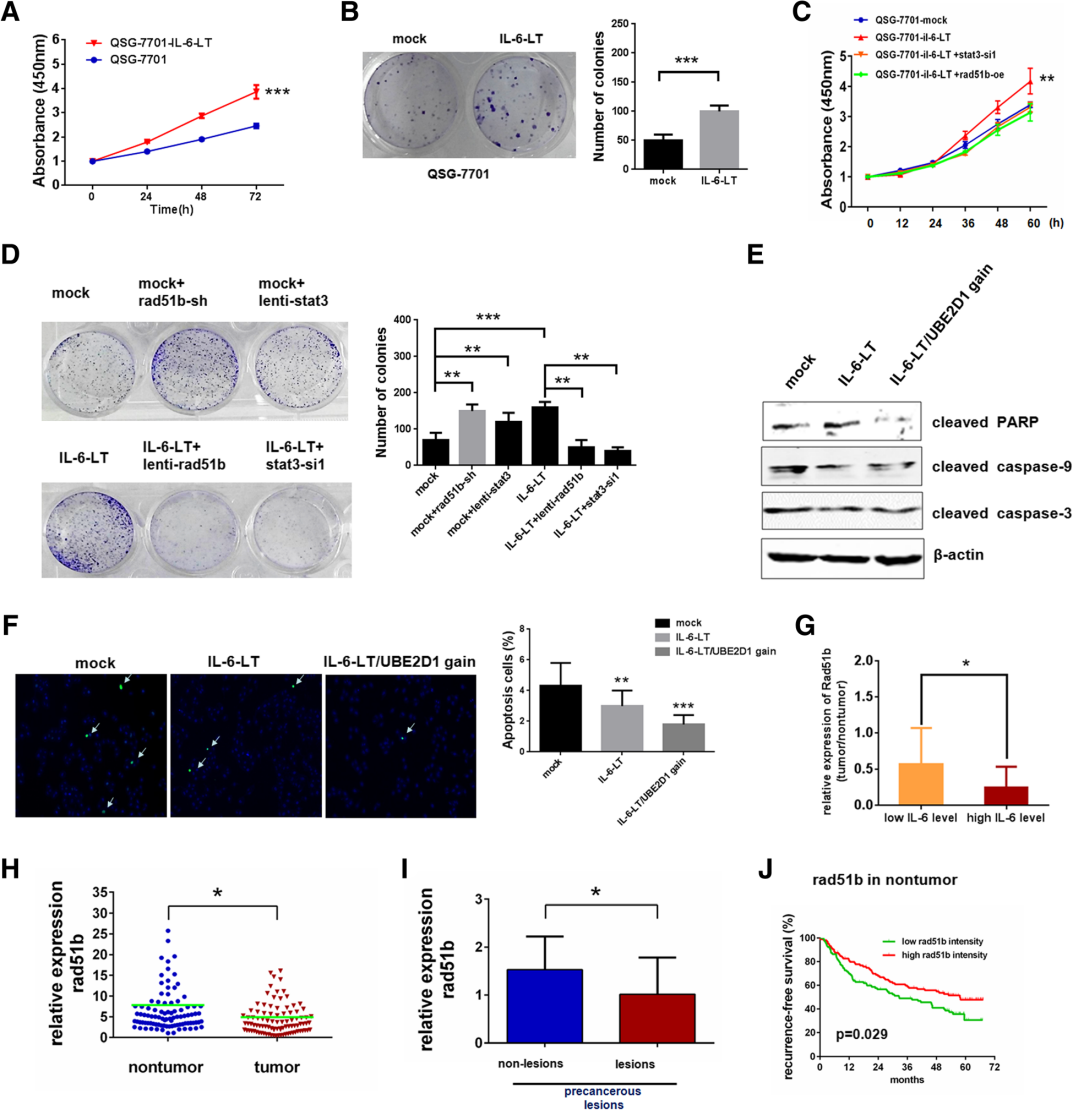
Functional roles of IL-6-RAD51B-UBE2D1 axis in HCC. **a** Cell numbers in continuous IL-6 treated QSG-7701 were determined by CCK-8 assay, and the relative number of cells is shown in mean ± standard error. **b** Plate clone formation assays for QSG-7701 cells treated with continuous IL-6. The number of colonies was showed in the right panel. **c** and **d** Cell proliferation by CCK-8 assays (**c**) and clone formation assays (**d**) to evaluate the roles of STAT3 and RAD51B under continuous IL-6 pressure. **e** and **f** Apoptosis in indicated QSG-7701 cells by Western blotting of apoptosis markers (**e**) or TUNEL assays (**f**). IL-6-LT/UBE2D1 gain indicated cell clones with UBE2D1 gains after 4 months IL-6 stimulation. IL-6-LT here indicated cell clones without UBE2D1 gains after 4 months IL-6 stimulation. The value of TUNEL score was showed in the right panel. **g** The relative expression of RAD51B in HCC patients with high serum IL-6 and low serum IL-6. y, the relative expression of RAD51B in HCC compared with nontumor tissues. x, the median intensity was used as the cutoff for low and high group. Independent-Sample T test was adopted for the statistical analysis. **h** The transcript level of RAD51B were determined by real-time PCR in HCC and paired adjacent nontumor tissues in HCC Cohort (*n* = 108). β-actin was used as internal control. **i** Relative expression of RAD51B in precancerous lesions and paired nontumor tissues without lesions(*n* = 9). Wilcoxon signed-rank test was adopted for the statistical analysis. **j** Kaplan-Meier analyses of the correlations between relative intensity of RAD51B in nontumor tissues in GSE14520 and recurrence-free survival of HCC patients. The median intensity was used as the cutoff for low and high group. *p < 0.05,**p < 0.01, ***p < 0.001

Next, we explored the relationship between continuous IL-6 and UBE2D1 copy number gain in HCC carcinogenesis. As continuous IL-6 played a different role from short-term stimulation in cancer [33], we first explored the effects of continuous IL-6 in HCC. We found that continuous IL-6 significantly facilitated the cell proliferation (Fig. 6a and Additional file 1: Figure S5A), clone formation potential (Fig. 6b and Additional file 1: Figure S5B) and tumor growth in vivo(Additional file 1: Fig. S5C). Additionally, interfering the STAT3 or restoring the RAD51B expression in continuous IL-6 treated cells would rescue the cell proliferation rate and clone formation potential (Fig. 6c, d).

As continuous IL-6 could increase the genomic content of UBE2D1 in part of HCC cells, we next explored the significance of UBE2D1 amplification under IL-6. Results showed that cells harboring UBE2D1 amplification under IL-6 enhanced the UBE2D1 protein level, while decreased p53 protein level, and exhibited reduced cell apoptosis and senescence phenotype than cells without UBE2D1 genomic alterations under IL-6 (Fig. 6e, f and Additional file 1: Figure S5D-F), indicating that UBE2D1 amplification enhanced the HCC promoting effects of continuous IL-6 stimulation.

Finally, we investigated the clinical significance of IL-6-RAD51B-UBE2D1 axis in HCC. By detecting the expression of RAD51B in the HCC cohort and precancerous lesions, we found that RAD51B was downregulated in both tumor tissues and precancerous lesions (Fig. 6g, h). Moreover, the transcript level of RAD51B was also associated with the serum IL-6 level of HCC patients, and patients with high serum IL-6 concentration had a lower RAD51B level (Fig. 6i). Low RAD51B expression in adjacent nontumor tissues suggested a poor recurrence-free survival of HCC patients (Fig. 6j). And STAT3 transcript level was negatively correlated with RAD51B in GSE14520(Additional file 1: Figure S5G).

## Discussion

HCC is a complicated disease with increasing incidence and mortality caused by cancer. Until now, sorafenib is the only choice for advanced stage HCC patients, but inherent or acquired drug resistance makes it invalid in some patients. So diagnosis and effective target therapy at early stage of HCC is an urgent task. In this study, we found that UBE2D1 was significantly upregulated in HCC tissues and high expression of UBE2D1 served as a predictor for poor prognosis of HCC patients. Moreover, UBE2D1 was also overexpressed in precancerous lesions and was upregulated only in cancer cells but not in hepatitis cells, indicating UBE2D1 as a specific HCC marker in early stage. One of the crucial events early in hepatocarcinogenesis has been proved to be copy number variations, which can control the gene expressions to initiate cancers [25]. We have demonstrated that UBE2D1 was frequently amplified in HCC and genomic copy number gain of UBE2D1 was correlated with the upregulation in HCC. More importantly, the genome content of UBE2D1 was also higher in precancerous lesions and HCC tissues but not in hepatitis tissues, suggesting that genomic alteration of UBE2D1 may play an important role in carcinogenesis of HCC. Previous studies have reported that exogenous UBE2D1 could interact with MDM2 to trigger the p53 ubiquitination in vitro as an E2s [7, 9]. However, the biological functions of UBE2D1 in cancers were unclear. We demonstrated that UBE2D1 could facilitate the cell proliferation, inhibit cell apoptosis and promote tumor growth in p53 wild type cells but not p53 mutated or deficient cells. The results indicated that UBE2D1 was also an important therapeutic target depending on p53.

HCC is an inflammatory cancer with significant gender disparity [2]. Correlation analyses and experiments in mice models confirmed the crucial roles of IL-6 in gender disparity of HCC. For example, serum IL-6 was an independent risk predictor for HCV-related HCC development in females [28], and reduced serum IL-6 concentration by Estrogens inhibits the diethylnitrosamine (DEN)-induced hepatocarcinogenesis in rodent models [27]. Our results showed that the genomic level of UBE2D1 was also associated with the gender of HCC patients, and males had a higher genomic level of UBE2D1 than females. Continuous IL-6 is reported to be produced by HCC progenitor cells in precancerous lesions in an acquired autocrine manner, and maintains in the malignant HCC [34]. In our study, we found that UBE2D1 genomic amplification was also observed in precancerous lesions, as well as advanced HCC tissues, and was correlated with the serum IL-6 level of HCC patients. Moreover, continuous IL-6 stimulation would facilitate the UBE2D1 genomic content in a certain proportion in cultured cells. Our results suggested the relationship between IL-6 and genomic alterations in HCC initiation via activating the DNA damage response and genomic instability, indicating a novel pattern to promote cancers of continuous IL-6 by inducing the genomic alterations of oncogenes. However, why region containing UBE2D1 was a specific fragment amplified under continuous IL-6 need further study. One reason may be that UBE2D1 located at a common fragile site(FRA10C), which is liable to generate rearrangements under replication stress [35].

Our results also indicated the important role of RAD51B in the continuous IL-6 stimulation to promote HCC carcinogenesis and growth. It has been reported that the haploinsufficiency or RNA interference-mediated knockdown of RAD51B would lead to centrosome fragmentation and chromosome instability [36] and RAD51B deficient DT40 cells exhibit an increase in chromosome aberrations [37]. Interestingly, other studies have reported that SNP in RAD51B was significantly associated with breast cancer risk in males [38], suggesting that RAD51B may be a common key factor in gender associated cancers. Our experiments demonstrated that UBE2D1 can promote HCC progression and genomic gain of UBE2D1 was attributed to IL-6/RAD51B axis.

## Conclusions

We found that UBE2D1 was upregulated in HCC and associated with poor prognosis of HCC patients. UBE2D1 could facilitate HCC growth in vitro and in vivo by enhancing the ubiquitin of p53. Genomic copy number gain of UBE2D1 contributed to its overexpression in HCC and was associated with high IL-6 level. The IL-6/RAD51B/UBE2D1 axis represents a new pattern of cytokines to drive genomic alterations in HCC initiation.

## Additional file

Additional file 1: **Figure S1.** UBE2D1 is upregulated in HCC. **Figure S2.** Molecular Characters of UBE2D1 in HCC and UBE2D1 promoted HCC growth. **Figure S3**. P53 mediated the pro-tumor effect of UBE2D1. **Figure S4.** Continuous IL-6 activated the DNA damage and genomic instability through Rad51b. **Figure S5.** Functional roles of IL-6-RAD51B-UBE2D1 axis in HCC. **Table S1.** Clinical Characteristics of the HCC Patients. **Table S2.** Clinical Characteristics of the Patients who provided hepatitis liver and normal liver tissues. **Table S3.** Oligonucleotides sequence. **Table S4.** Correlation of UBE2D1 genomic level with gender in HCC. **Table S5.** Serum IL-6 concentration of HCC patients. Experimental Procedures. (DOC 1472 kb)

## Abbreviations

CCK-8: Cell Counting Kit-8
CNVs: Copy number variations
Co-IP: Co-immunoprecipitation
HCC: Hepatocellular carcinoma
IHC: Immunohistochemistry
RAD51B: RAD51 Paralog B
UBE2D1: Ubiquitin Conjugating Enzyme E2 D1
