# Supplementary material for: Gain of UBE2D1 facilitates hepatocellular carcinoma progression and is associated with DNA damage caused by continuous IL-6
Source: J Exp Clin Cancer Res. 2018 Nov 27;37:290. doi: 10.1186/s13046-018-0951-8 (PMC6260761; doi:10.1186/s13046-018-0951-8)
Supplement: Supplementary file 1 — Figure S1. UBE2D1 is upregulated in HCC. Figure S2. Molecular Characters of UBE2D1 in HCC and UBE2D1 promoted HCC growth. Figure S3. P53 mediated the pro-tumor effect of UBE2D1. Figure S4. Continuous IL-6 activated the DNA damage and genomic instability through Rad51b. Figure S5. Functional roles of IL-6-RAD51B-UBE2D1 axis in HCC. Table S1. Clinical Characteristics of the HCC Patients. Table S2. Clinical Characteristics of the Patients who provided hepatitis liver and normal liver tissues. Table S3. Oligonucleotides sequence. Table S4. Correlation of UBE2D1 genomic level with gender in HCC. Table S5. Serum IL-6 concentration of HCC patients. Experimental Procedures. (DOC 1472 kb) [file 13046_2018_951_MOESM1_ESM.doc]

**SUPPLEMENTARY DATA**

**Gain of UBE2D1 facilitates hepatocellular carcinoma progression and is associated with DNA damage caused by continuous IL-6**

Chuanchuan Zhou, Fengrui Bi, Jihang Yuan, Fu Yang, and Shuhan Sun

**Inventory**

**Supplemental Data**

**Figure S1.** UBE2D1 is upregulated in HCC.

**Figure S2.** Molecular Characters of UBE2D1 in HCC and UBE2D1 promoted HCC growth.

**Figure S3. P53 mediated the pro-tumor effect of UBE2D1.**

**Figure S4.** Continuous IL-6 activated the DNA damage and genomic instability through Rad51b.

**Figure S5.** Functional roles of IL-6-RAD51B-UBE2D1 axis in HCC.

**Table S1.** Clinical Characteristics of the HCC Patients.

**Table S2.** Clinical Characteristics of the Patients who provided hepatitis liver and normal liver tissues.

**Table S3.** Oligonucleotides sequence.

**Table S4.** Correlation of UBE2D1 genomic level with gender in HCC.

**Table S5.** Serum IL-6 concentration of HCC patients.

**Supplemental experimental procedures**


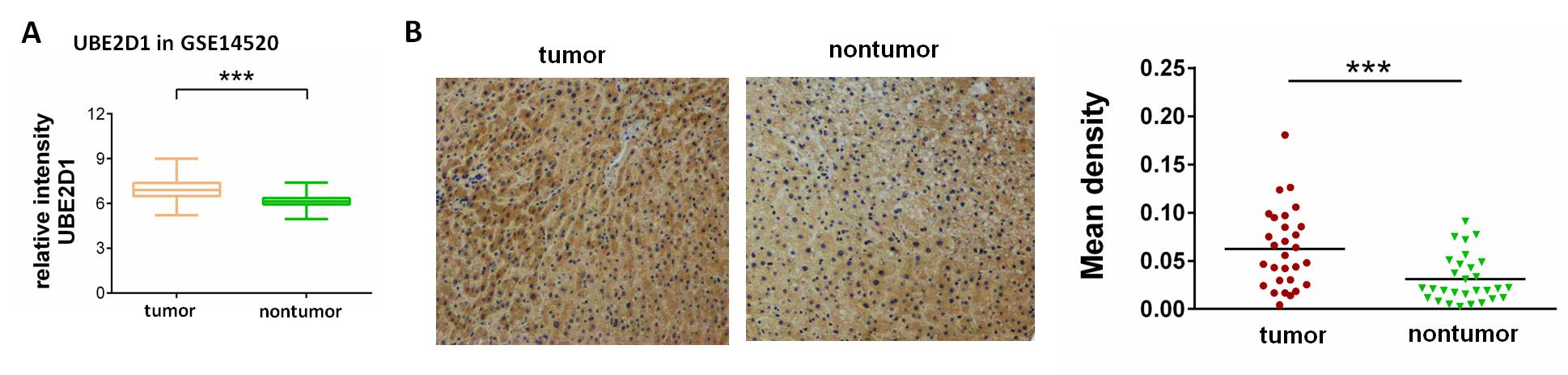


**Figure S1. UBE2D1 was upregulated in HCC.** (**A**) Relative intensity of UBE2D1 in HCC and compared noncancerous liver tissues in GSE14520. **(B)** Representative immunohistochemistry image of UBE2D1 in HCC compared with nontumor tissues(n=30, from the same set of Cohort). The value of immunohistochemistry score was showed in the right panel. ***p<0.001.


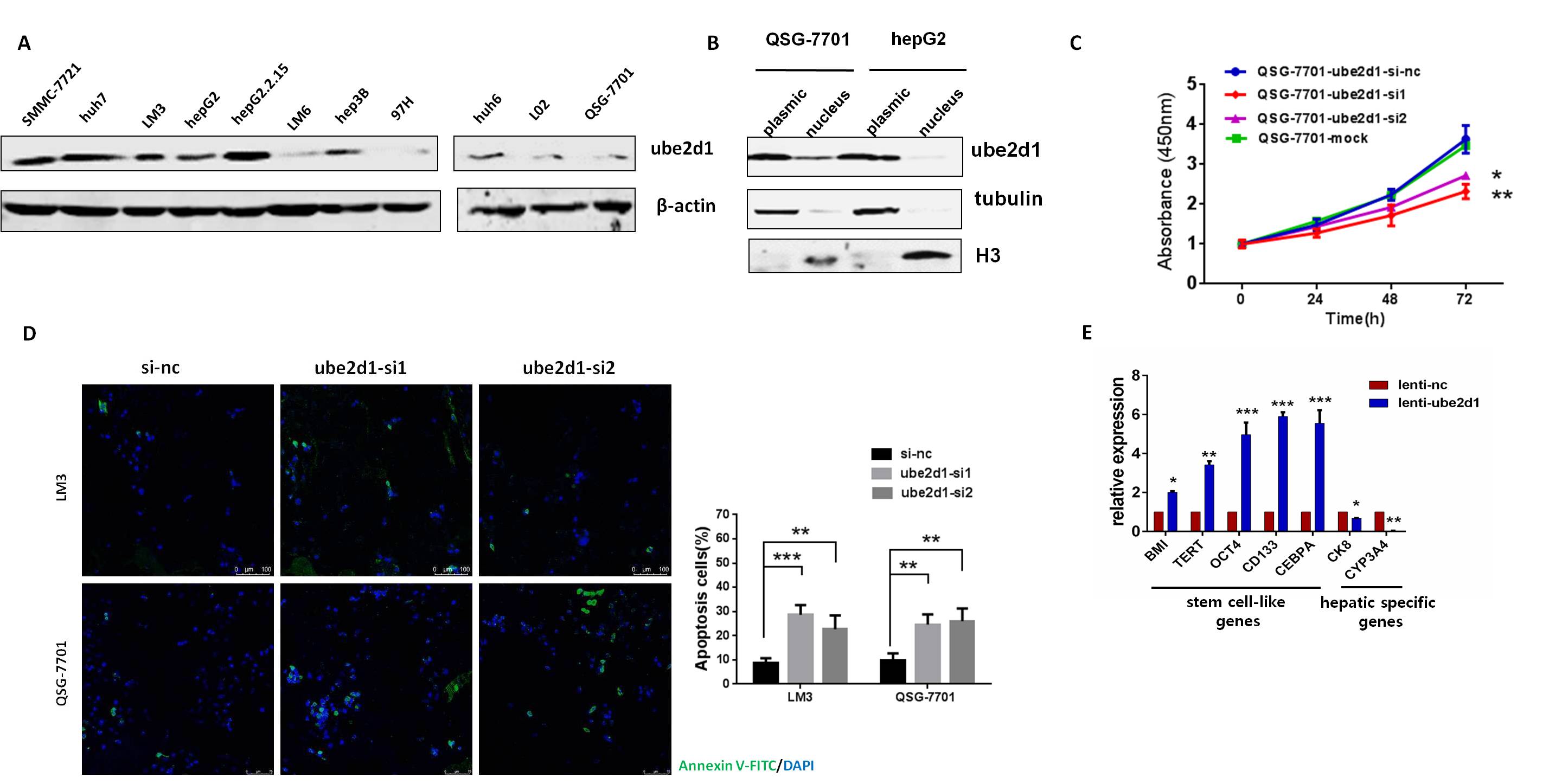


**Figure S2. Molecular Characters of UBE2D1 in HCC and UBE2D1 promoted HCC growth.** **(A)** Protein level of UBE2D1 in serious of hepatic cell lines(L02 and QSG7701) and HCC cell lines(others). **(B)** The protein levels of UBE2D1 by western blot in nucleus and cytoplasmic protein extraction. Tubulin was used as the cytoplasmic specific control, and Histone H3 was used as the nucleus specific control. **(C)** CCK-8 assays for cell proliferation in UBE2D1 silent QSG-7701 cells. mock, cells without siRNA-transfection.  **(D)** Annexin V-FITC staining in UBE2D1 silent LM3 and QSG-7701 cells. The nuclei were stained by DAPI. The number of apoptosis cells was showed in the right panel. **(E)** Relative expression of stem cell-like genes and hepatic specific genes to evaluate the function of UBE2D1 in pluripotency. *p<0.05, **p<0.01, ***p<0.001.

###
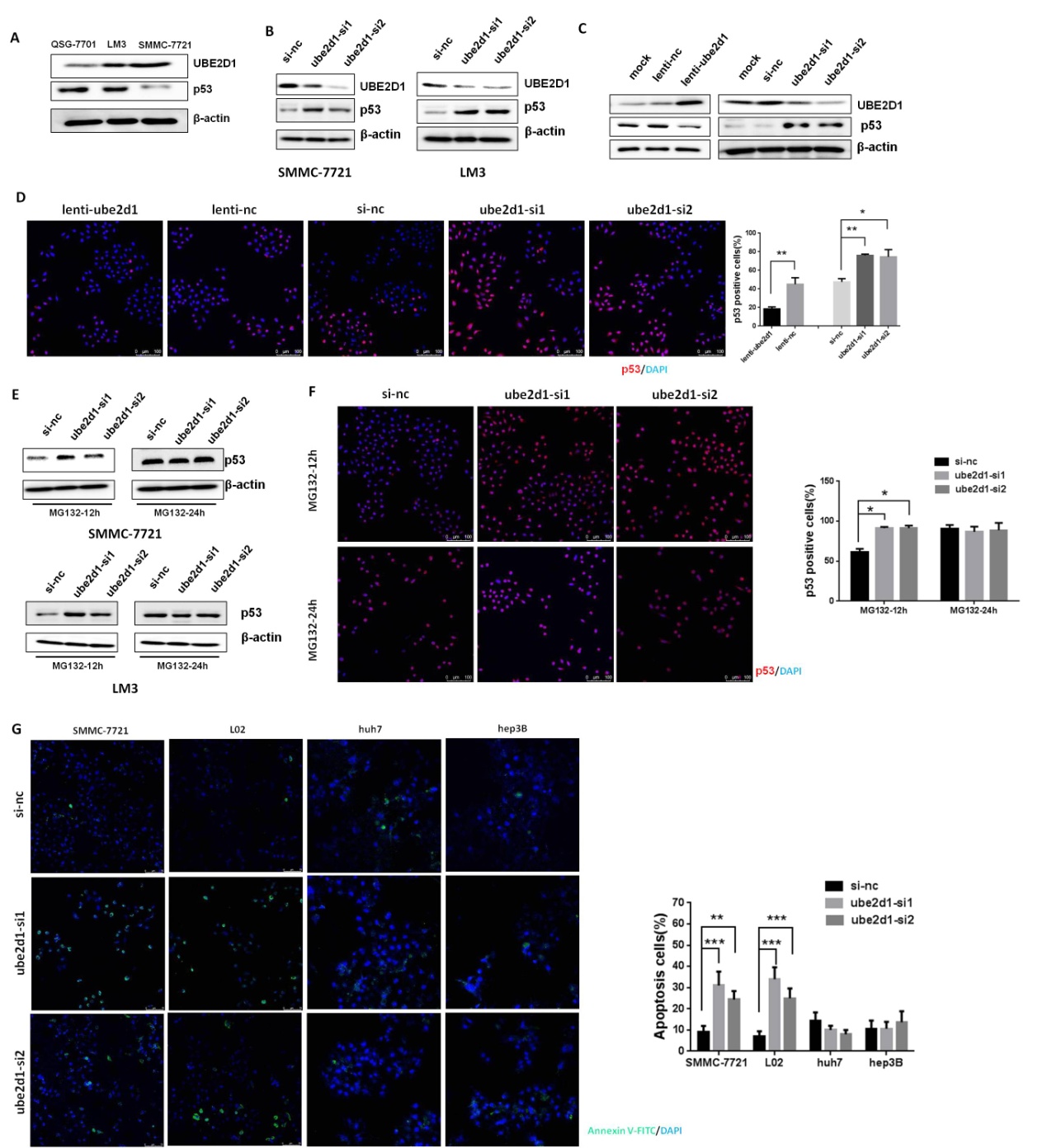


**Figure S3. P53 mediated the pro-tumor effect of UBE2D1. (A)** p53 protein level determined by western blot in QSG-7701, LM3 and SMMC-7721 cells. **(B)** UBE2D1 and p53 protein level determined by western blot in UBE2D1 underexpressed SMMC-7721 and LM3 cells. **(C)** UBE2D1 and p53 protein level determined by western blot in UBE2D1 overexpressed and underexpressed QSG-7701 cells. mock, cells without siRNA-transfection or lentivirus-infected. **(D)** p53+ nuclei in UBE2D1 overexpressed and underexpressed QSG-7701 cells were determined by immunofluorescence. The number of p53 positive cells was showed in the right panel. **(E)** P53 western blot assays in UBE2D1 silent and negative control SMMC-7721 and LM3 cells after treated with MG132 for 12h and 24h. **(F)** p53+ nuclei were determined by immunofluorescence in UBE2D1 underexpressed QSG-7701 cells after treated with MG132 for 12h and 24h. The number of p53 positive cells was showed in the right panel. **(G)** Annexin V-FITC staining in UBE2D1 silent SMMC-7721, L02, huh7 and hep3B cells. The nuclei were stained by DAPI. The number of apoptosis cells was showed in the right panel.*p<0.05, **p<0.01, ***p<0.001.

###
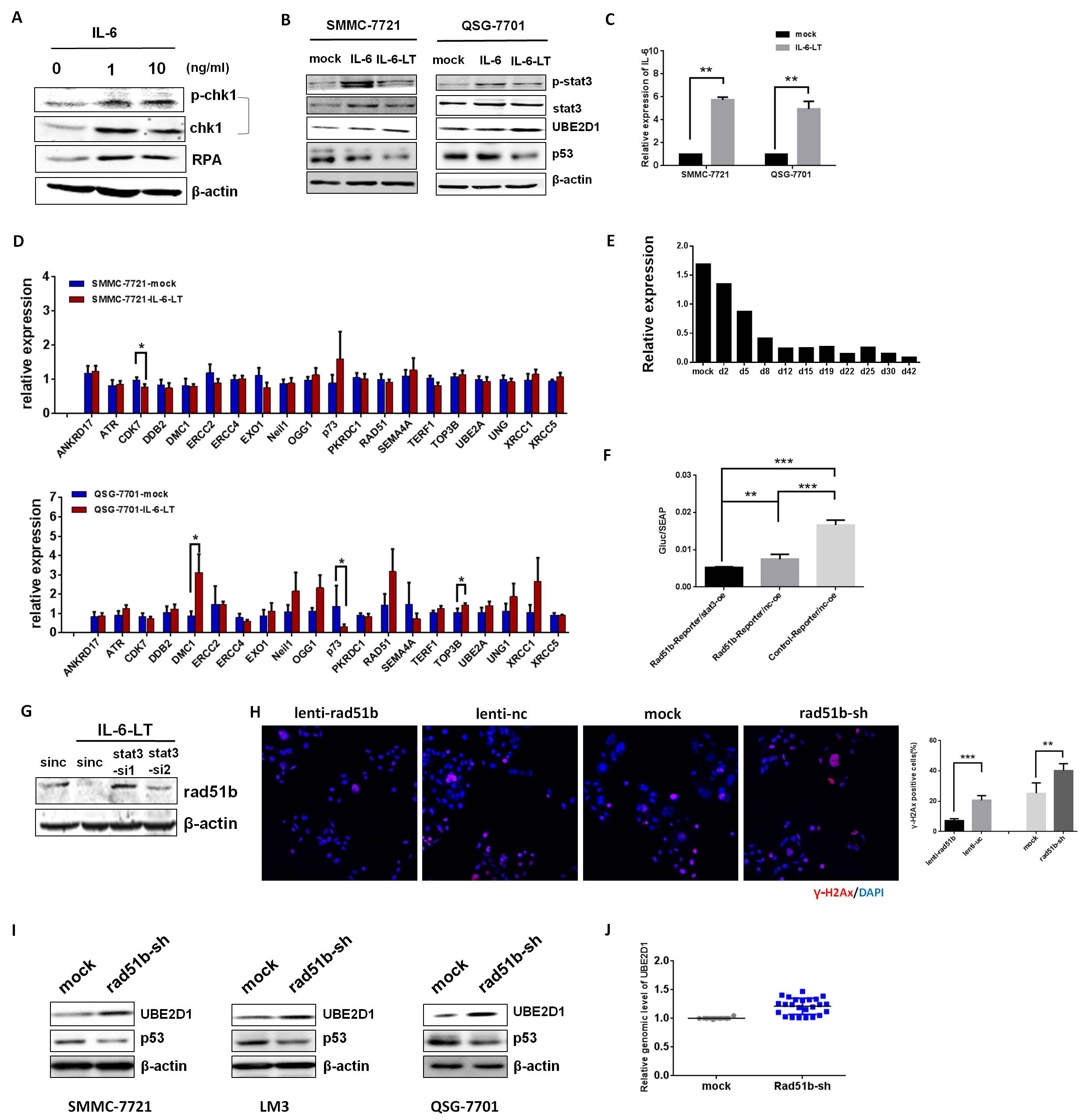


**Figure S4. Continuous IL-6 activated the DNA damage and genomic instability through Rad51b. (A)** DNA damage markers determined by western blot in QSG-7701 cells treated with indicated concentration of IL-6 for 24h. **(B)** Detection of STAT3, p-STAT3, UBE2D1, p53 in continuous IL-6 (more than 2 months) treated QSG-7701 and SMMC-7721 cells. **(C)** Expression of IL-6 in continuous IL-6 treated QSG-7701 and SMMC-7721 cells measured by qRT-PCR. **(D)** Relative expression of other candidate DNA damage repair associated genes in continuous IL-6 treated SMMC-7721 and QSG-7701 cells. **(E)** Relative expression of Rad51b under IL-6 at indicate time in QSG-7701 cells. d, indicated days. **(F)** Results of suppressive effect of STAT3 on the Rad51b promoter measured by Rad51b reporter assay.  STAT3 overexpression vector(STAT3-oe) or blank vector(nc-oe) was co-transfected with Rad51b promoter-luciferase reporter plasmids or negative control reporter plasmids. GLuc activity was determined 48h after transfection and the Gluc activity was normalized by SEAP activity. **(G)** Protein levels of Rad51b in STAT3 silent and control SMMC-7721 cells with continuous IL-6. **(H)** γ-H2Ax foci in Rad51b overexpressed and silent cells were determined by immunofluorescence. Nuclei were stained by DAPI. The number of γ-H2Ax foci positive cells was showed in the right panel and data was collected from three independent experiments. **(I)** UBE2D1 and p53 protein level measured by Western blotting in Rad51b stably underexpressed cells. **(J)** Genomic levels of UBE2D1 were detected by real-time PCR in Rad51b stably underexpressed SMMC-7721 cells for more than 4 months.*p<0.05,**p<0.01, ***p<0.001.


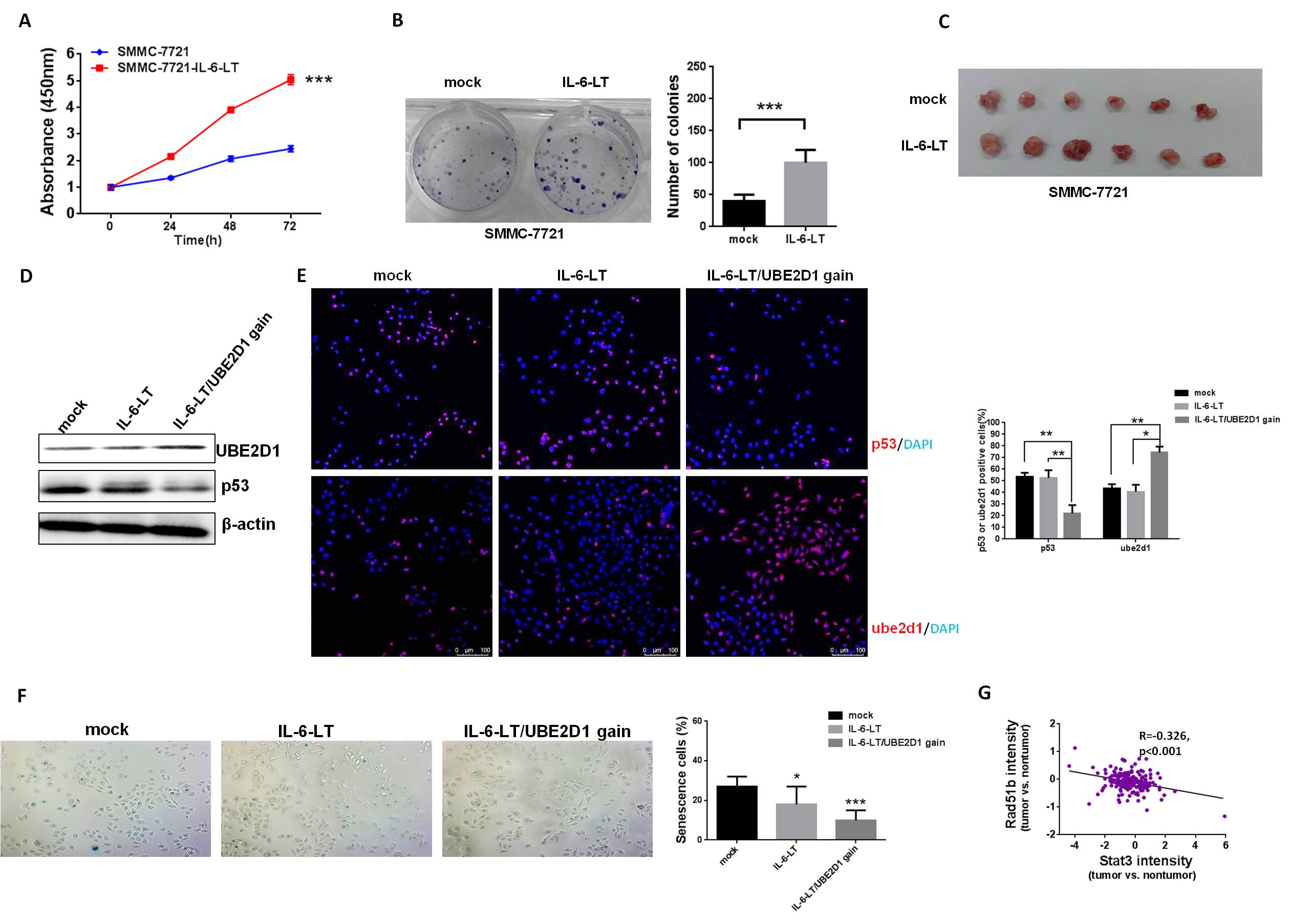


**Figure S5. Functional roles of IL-6-RAD51B-UBE2D1 axis in HCC. (A)** Cell numbers in continuous IL-6 treated SMMC-7721 were determined by CCK-8 assay, and the relative number of cells is shown in mean ±standard error. **(B)** Plate clone formation assays for SMMC-7721 cells treated with continuous IL-6. The number of colonies was showed in the right panel. **(C)** Tumors from nude mice with 1 X 10^5^ indicated cells injected subcutaneously in bilateral armpit. n=6 for each group. **(D)** UBE2D1 and p53 protein level measured by western blotting in continuous IL-6 treated cells. IL-6-LT/UBE2D1 gain indicated cell clones with UBE2D1 gains after 4 months IL-6 stimulation. IL-6-LT here indicated cell clones without UBE2D1 gains after 4 months IL-6 stimulation. **(E)** UBE2D1 and p53 protein were determined by immunofluorescence in continuous IL-6 treated cells. IL-6-LT/UBE2D1 gain indicated cell clones with UBE2D1 gains after 4 months IL-6 stimulation. IL-6-LT here indicated cell clones without UBE2D1 gains after 4 months IL-6 stimulation. The number of UBE2D1 or p53 positive cells was showed in the right panel. **(F)** Senescence phenotype detected by b-galactosidase (SA-b-gal) staining in indicated QSG-7701 cells. IL-6-LT/UBE2D1 gain indicated cell clones with UBE2D1 gains after 4 months IL-6 stimulation. IL-6-LT here indicated cell clones without UBE2D1 gains after 4 months IL-6 stimulation. **(G)** Correlation of STAT3 and RAD51B transcript level in HCC in GSE14520. x, the STAT3 intensity in HCC tissues compared with noncancerous tissues; y, the Rad51b intensity in HCC tissues compared with noncancerous tissues. *p<0.05, **p<0.01, ***p<0.001.

**Table S1**. Clinical Characteristics of the HCC Patients.

| Variable | Cohort (n=108) |
| --- | --- |
| Sex — no. |  |
| Female  Male | 33  75 |
| Tumor size — no. |  |
| ≤5 cm | 45 |
| >5 cm | 63 |
| Multinodular tumor |  |
| Yes | 36 |
| No | 72 |
| Cirrhosis — no. |  |
| Yes | 60 |
| No | 48 |
| BCLC stage — no. |  |
| 0 | 3 |
| A | 70 |
| B | 22 |
| C | 13 |
| Capsule |  |
| No | 19 |
| Intact | 17 |
| Not intact | 72 |
| Microvascular Invasion |  |
| Yes | 47 |
| No | 61 |

**Table S2. Clinical Characteristics of** the Patients who provided hepatitis liver and normal liver tissues.

| Variable | normal (n=41) | hepatitis (n=22) |
| --- | --- | --- |
| Sex — no. |  |  |
| Female | 28 | 12 |
| Male | 13 | 10 |
| Hepatitis B virus — no. |  |  |
| Negative | 41 | 0 |
| Positive | 0 | 22 |
| Cirrhosis — no. |  |  |
| Yes | 1 | 1 |
| No | 40 | 21 |
| Clinical diagnosis | Liver cavernous hemangioma | hepatitis B virus infection |

**Table S3. Oligonucleotides sequence**

| Primer names | Sequences |
| --- | --- |
| UBE2D1(RT-PCR) | GGACCTGTGGGAGATGACTTG |
|  | TGGTACTAAGGGGTCATCTGGA |
| UBE2D1(gDNA-PCR) | AGGAAGAGAAACCGCACCAG |
|  | GAGGGTGTGACAGGGAAAGG |
| LINE-1 | AAAGCCGCTCAACTACATGG |
|  | TGCTTTGAATGCGTCCCAGAG |
| β-actin | TGTGTTGGCGTACAGGTCTTTG |
|  | GGGAAATCGTGCGTGACATTAAG |
| STAT3 | ACTTCAGACCCGTCAACAAAT |
|  | GCTCCACCACAAAGGCAC |
| RAD51B | AGCAAAGGAAGCGAAAT |
|  | CGTGGAAACAGGAGGTG |
| p53 | TTGAGGTGCGTGTTTGTG |
|  | CTGGGCATCCTTGAGTTC |
| BMI | ATGTATGAGGAGGAACC |
|  | CAAACAAGAAGAGGTGGA |
| TERT | CAGAGGTCAGGCAGCATC |
|  | CGCCCGCTCGTAGTTGA |
| OCT4 | GAGAAAGCGAACCAGTATC |
|  | CCTGAGAAAGGAGACCC |
| CD133 | CTCTATACCAAAGCGTCAA |
|  | CGATGCCACTTTCTCAC |
| CEBPA | TTGGTGCGTCTAAGATGAGGG |
|  | GCATTGGAGCGGTGAGTTTG |
| CK8 | GCCGTGGTTGTGAAGAAGAT |
|  | CTCCTGTTCCCAGTGCTAC |
| CYP3A4 | CCGAGTGGATTTCCTTCAG |
|  | GGTGGTGCCTTATTGGGTA |
| IL-6 | GGAGACTTGCCTGGTGAA |
|  | GCATTTGTGGTTGGGTCA |
| UBE2D1-si-1 sense  UBE2D1-si-1 antisense | 5’-GACUCCUGAUAGCGCAUAUTT-3’  5’-AUAUGCGCUAUCAGGAGUCTT-3’ |
| UBE2D1-si-2 sense  UBE2D1-si-2 antisense | 5’-CCAGCUCUGACUGUAUCAATT-3’  5’-UUGAUACAGUCAGAGCUGGTT-3’ |
| STAT3-si-1 sense  STAT3-si-1 antisense | 5’-CCCGGAAAUUUAACAUUCUTT-3’  5’-AGAAUGUUAAAUUUCCGGGTT-3’ |
| STAT3-si-2 sense  STAT3-si-2 antisense | 5’-GGGACCUGGUGUGAAUUAUTT-3’  5’-AUAAUUCACACCAGGUCCCTT-3’ |
| RAD51B-chip qPCR sense  RAD51B-chip qPCR antisense | 5’- ACCGACTCTATCTTGCTTCTAAC -3’  5’- TTTCGGAAAGGGCTGTTAT -3’ |

**Table S4.** Correlation of UBE2D1 genomic level with gender in HCC

| **gender** | **UBE2D1 genomic level in HCC tissues** | | | **total patients** | **p value** |
| --- | --- | --- | --- | --- | --- |
|  | **low(36)** | **media(36)** | **high(36)** |  |  |
| male | 19 | 28 | 28 | 75 | 0.029 |
| female | 17 | 8 | 8 | 33 |  |

**Table S5. Serum IL-6 concentration of HCC patients**

|  | **Low IL-6 group(n=32)*** | **High IL-6 group(n=32)*** |
| --- | --- | --- |
| **IL-6 concentration** **(pg/ml)** | 27.225 | 67.086 |
|  | 30.500 | 68.774 |
|  | 33.314 | 68.774 |
|  | 39.667 | 69.498 |
|  | 41.556 | 71.190 |
|  | 42.974 | 71.673 |
|  | 43.210 | 71.673 |
|  | 45.104 | 72.157 |
|  | 45.578 | 72.157 |
|  | 45.815 | 74.579 |
|  | 47.475 | 77.005 |
|  | 48.900 | 78.463 |
|  | 51.755 | 78.950 |
|  | 51.993 | 83.091 |
|  | 51.993 | 83.824 |
|  | 52.231 | 84.068 |
|  | 52.469 | 84.556 |
|  | 53.184 | 84.801 |
|  | 53.661 | 87.002 |
|  | 54.854 | 89.697 |
|  | 54.854 | 90.679 |
|  | 56.526 | 91.415 |
|  | 56.526 | 93.382 |
|  | 57.243 | 93.382 |
|  | 57.722 | 96.584 |
|  | 59.158 | 99.546 |
|  | 59.158 | 111.217 |
|  | 61.795 | 112.715 |
|  | 64.197 | 120.983 |
|  | 65.400 | 131.584 |
|  | 66.122 | 163.927 |
|  | 66.845 | 288.355 |

***** Serum IL-6 concentration was acquired by ELISA. The median intensity was used as the cutoff for low and high group.

**Supplemental Experimental Procedures**

**Nucleotide extraction and real-time PCR**

Total RNA was extracted with TRIzol reagent (Invitrogen) and first-strand cDNA was synthesized with the PrimeScript RT reagent Kit (TaKaRa, Dalian, China). Quantitative real-time PCR (RT-PCR) for the transcript level was performed using SYBR Green (Takara, Dalian, China) according to a standard protocol in the StepOne Plus system (Applied Biosystems, Foster City, CA). β-actin was used as internal control. Genomic DNA content was prepared according the DNA extraction procedure using AxyPrep Genomic DNA Miniprep Kit (Axygen Biosciences, CA, USA). Real-time PCR for genomic DNA was performed as above. LINE-1(Longinterspersed nuclearelement-1) were used as internal control as indicated. Using LINE-1 as the inner control, a repetitive element for which copy numbers per haploid genome are similar among all of the human normal and tumor cells, the genomic level of specific gene can be determined by Real-time PCR. The gene-specific primers are shown in Supplementary Table S3.

**Cytoplasmic and nuclear protein extraction**

The isolation of cytoplasmic and nuclear proteins were performed followed the manufacturer’s instruction of the ProteoJETTM Cytoplasmic and Nuclear Protein Extraction Kit (Fermentas, St. Leon-Rot, Germany).

**SA-b-gal staining for senescence detection**

We performed the cytochemical staining for b-galactosidase assay to detect the senescence-associated phenotype of indicated cells. Cells were cultured in 24-well plate at the cell density of 1 × 10^3^ cells/well. Fixed with 3.7% formaldehyde, the cells were incubated overnight at 37°C without CO2 in a staining reaction components according to the protocol provided by the b-galactosidase staining kit (Beyotime Biotechnology, China). The images of cell morphology were captured under the Olympus microscopy (IX71; Olympus, Japan).

**Chromatin immunoprecipitation (ChIP)**

To examine the binding of phosphorylated STAT3 in the promoter of RAD51B, we performed the ChIP with EZ ChIP Chromatin Immunoprecipitation Kit (Millipore, Bedford, MA, USA). We sonicated the crosslinked chromatin into 200- to 500-bp fragments, and immunoprecipitated the target DNA sequences using a specific antibody to phosphorylated STAT3. IgG was used as negative control. We then performed real-time PCR to examine the enrichment of Rad51b promoter. The primers was designed to the predict site in the promoter of RAD51B using programs in the gene-regulation( <http://www.gene-regulation.com/pub/programs.html>). The primer sequences are listed in Supplementary Table S3.

**Co-Immunoprecipitation (Co-IP)**

For total cell extracts, cells were lysed in buffer containing 50 mM Tris-HCl (pH 7.5), 150 mM NaCl, 1% Triton X-100 and cleared by centrifugation. Immunoprecipitation with specific antibodies to MDM2(Sigma-Aldrich) were performed as the protocol of Pierce Co-Immunoprecipitation Kit (Thermo, Rockford, USA) described. The purified protein components were then analyzed by western blotting with specific antibodies to p53. The immunoprecipitation of lgG was also performed as negative controls.

**Annexin V-FITC staining**

For the detection of apoptosis, Annexin V-FITC staining was performed according to the manufacturer's instruction (sangon, shanghai, China). Cells were cultured in slides in 12-well plate. After washing slides by PBS and binding solution, cells were incubated with Annexin V-FITC reaction buffer for 30min. Nuclei was stained by DAPI, and images were captured under the Olympus microscopy (IX71; Olympus, Japan).

**Luciferase reporter assay**

293T cells cultured in 12-well plate. 250ng of STAT3 overexpression vector(STAT3-oe) or blank vector(nc-oe) was co-transfected with 250ng Rad51b promoter-luciferase reporter plasmids or negative control reporter plasmids. Cell cultured medium was refreshed 24h after transfection, and was collected another 24h. 10ul medium was mixed with 100ul of Gluc substrate solution to get the Gluc activity value. Secreted alkaline phosphatase (SEAP) in the same reporter vector was used for normalization. Another 10ul of the same medium was mixed with 100ul of SEAP substrate solution to get the SEAP activity value. The suppressing effect of STAT3 on Rad51b promoter was evaluated by ratio of Gluc activity value and SEAP activity value.

Supplementary reference

1. Wang TL, Maierhofer C, Speicher MR, Lengauer C, Vogelstein B, Kinzler KW, Velculescu VE: **Digital karyotyping**. *Proceedings of the National Academy of Sciences of the United States of America* 2002, **99**(25):16156-16161.

2. Moroni M, Veronese S, Benvenuti S, Marrapese G, Sartore-Bianchi A, Di Nicolantonio F, Gambacorta M, Siena S, Bardelli A: **Gene copy number for epidermal growth factor receptor (EGFR) and clinical response to antiEGFR treatment in colorectal cancer: a cohort study**. *The Lancet Oncology* 2005, **6**(5):279-286.

3. Zhao X, Li C, Paez JG, Chin K, Janne PA, Chen TH, Girard L, Minna J, Christiani D, Leo C *et al*: **An integrated view of copy number and allelic alterations in the cancer genome using single nucleotide polymorphism arrays**. *Cancer research* 2004, **64**(9):3060-3071.
